# Needle Clogging in Biostimulator Injection: Rheological Context and Clinical Experience

**DOI:** 10.1111/jocd.70713

**Published:** 2026-01-30

**Authors:** Jui‐Yu Lin, Chuan‐Yuan Lin

**Affiliations:** ^1^ Nanzi Annyeong Clinic Kaohsiung City Taiwan

**Keywords:** biostimulator injection, mesotherapy injector control device, microparticle aggregation, microparticle jamming, needle clogging, suspension rheology

To the Editor,

We read with great interest the recent article in the *Journal of Cosmetic Dermatology* entitled “Electron Microscopy–Based Study of Cannulas for Suspension‐Based Dermal Filler and Biostimulator Application.” Radke et al. [1] compared 22‐gauge blunt cannulas from two brands using electron microscopy, focusing on exit opening geometry and inner lumen surface features relevant to the risk of clogging during administration of suspension‐based injectables. We offer the following comments in the spirit of complementing the authors' important findings with additional rheological and clinical context.

In the article, biostimulators are described as generally behaving as Newtonian fluids [1]. However, from a rheological standpoint, they should not be classified as Newtonian. Rather, they represent non‐Newtonian particulate suspensions whose flow behavior is governed by microparticle–microparticle interactions, the properties of the suspending medium, and geometric confinement within needles or cannulas [2].

With respect to needle clogging, our clinical experience differs from the implication that clogging is common when using 22‐gauge cannulas. Numerous biostimulators are currently approved for clinical use in China, and clogging is seldom encountered when using 27‐ or 25‐gauge cannulas and is only very rarely observed with 23‐ or 22‐gauge cannulas. Most instances of clogging occur with lyophilized, carboxymethylcellulose‐based biostimulators, particularly Sculptra. When reconstitution is performed thoroughly and uniformly, the occurrence of clogging can be largely eliminated [3, 4].

Needle clogging is a multifactorial phenomenon. Prior research has shown that smaller needle inner diameter (ID), higher vehicle viscosity, and larger particle size significantly increase obstruction risk—findings consistent with our experience. Additional contributing factors are summarized in Table 1 [5]. Although Radke et al. cite a microparticle size range of 2–150 μm [1], most published reports indicate that the majority of commercially available biostimulators contain microparticles predominantly within the 20–80 μm range [6], which remains smaller than the ID of a 32‐gauge needle (Table 2). In practice, clogging may occur through two principal mechanisms. First, microparticle aggregation may arise, particularly in products containing carboxymethylcellulose that require reconstitution before injection. When reconstitution is incomplete, some microparticles may form clumps; if the size of such a clump exceeds the needle ID, mechanical obstruction results. Second, microparticle jamming may occur in small‐bore needles even when individual microparticles are smaller than the lumen. Near the needle orifice or within the lumen, interactions between multiple microparticles—mediated by wall‐to‐microparticle and microparticle‐to‐microparticle friction—may create transient or persistent packing structures that impede flow [3, 4].

**TABLE 1:** Major determinants of needle clogging during injection of suspension‐based biostimulators.

Determinant	Observed effect on clogging	Underlying mechanism	Clinical implication
Particle concentration (volume fraction)	Higher concentration markedly increases clogging	Local particle crowding and jamming at constrictions	Dilution reduces clogging risk
Needle inner diameter	Smaller inner diameter substantially increases clogging risk	Increased particle‐to‐orifice diameter ratio promotes bridging	Larger‐bore cannulas improve injectability
Vehicle viscosity	Higher viscosity increases clogging even at slower injection rates	Greater drag forces push particles into the contraction zone	Lower‐viscosity suspensions flow more reliably
Particle characteristics (size and shape)	Larger particles and non‐spherical particles increase clogging	Bridging and filter‐like stacking behavior	Smaller, spherical, uniform particles reduce risk
Injection/tissue environment	Backpressure and syringe design can convert partial jamming into full occlusion	Porous tissue acts as a particle filter; sudden‐taper hubs increase entrance blockage	Gradual‐taper hubs, avoiding forceful injection help mitigate risks

**TABLE 2:** Approximate inner diameters of commonly used hypodermic needles by gauge size.

Needle gauge	Approximate inner diameter (μm)
32‐gauge	108
31‐gauge	133
30‐gauge	159
29‐gauge	184
27‐gauge	210
25‐gauge	260
23‐gauge	337
22‐gauge	413

More recently, biostimulators have also been introduced for intradermal injection to improve skin texture. This technique commonly employs automated multi‐needle injectors with very small‐bore needles (e.g., 32‐gauge), under which conditions clogging is observed more frequently than in deeper injection planes [4]. Several measures may help mitigate this problem, including thorough and uniform reconstitution to eliminate microparticle clumps, use of lower‐concentration suspensions to reduce viscosity, and application of a mesotherapy injector control device (MICD; Hertz Bio., Shenzhen, China) to reduce the likelihood of microparticle jamming. The MICD continuously monitors subtle changes in extrusion resistance, injection speed, and negative pressure at the skin–probe interface and temporarily interrupts injection to relieve microparticle packing before resuming delivery. With appropriate reconstitution, optimization of suspension concentration, and MICD assistance, needle clogging during intradermal injection becomes uncommon.

In conclusion, needle clogging is a multifactorial event. Although needle geometry contributes to risk, key determinants include needle ID, particle size, and suspension concentration. While clogging is uncommon in routine practice, it may occur when reconstitution is incomplete or when intradermal delivery is performed through very small‐bore multi‐needle devices. Attention to reconstitution technique, viscosity optimization, and supportive delivery technologies may help reduce obstruction risk and improve procedural safety.

## Conflicts of Interest

The authors declare no conflicts of interest.

## Linked Articles

This article is linked to https://doi.org/10.1111/jocd.70572.

## Data Availability

The authors have nothing to report.
